# Diabetes Mellitus and Risk of Bladder Cancer: A Meta-Analysis of Cohort Studies

**DOI:** 10.1371/journal.pone.0056662

**Published:** 2013-02-20

**Authors:** Zhaowei Zhu, Xiaohua Zhang, Zhoujun Shen, Shan Zhong, Xianjin Wang, Yingli Lu, Chen Xu

**Affiliations:** 1 Department of Urology, Ruijin Hospital, Shanghai Jiaotong University School of Medicine, Shanghai, China; 2 Institute and Department of Endocrinology and Metabolism, Shanghai Ninth People’s Hospital, Shanghai Jiaotong University School of Medicine, Shanghai, China; 3 Department of Embryology and Histology, Shanghai Jiaotong University School of Medicine, Shanghai, China; 4 Shanghai Key Laboratory of Reproductive Medicine, Shanghai Jiaotong University School of Medicine, Shanghai, China; Tehran University of Medical Sciences, Iran (Republic of Islamic)

## Abstract

**Background:**

Increasing evidence suggests that diabetes mellitus (DM) may be associated with an increased risk of bladder cancer. To provide a quantitative assessment of this association, we evaluated the relation between DM and incidence and mortality of bladder cancer in an updated meta-analysis of cohort studies.

***Methods*** We identified cohort studies by searching the EMBASE and MEDLINE databases, through 31 March 2012. Summary relative risks (RRs) with 95% confidence intervals (CIs) were calculated with random-effects models.

**Results:**

A total of 29 cohort studies (27 articles) were included in this meta-analysis. DM was associated with an increased incidence of bladder cancer (RR 1.29, 95% CI: 1.08–1.54), with significant evidence of heterogeneity among these studies (p<0.001, I^2^ = 94.9%). In stratified analysis, the RRs of bladder cancer were 1.36 (1.05–1.77) for diabetic men and 1.28 (0.75–2.19) for diabetic women, respectively. DM was also positively associated with bladder cancer mortality (RR 1.33, 95% CI: 1.14–1.55), with evident heterogeneity between studies (p = 0.002, *I^2^* = 63.3%). The positive association was observed for both men (RR 1.54, 95% CI: 1.30–1.82) and women (RR 1.50, 95% CI: 1.05–2.14).

**Conclusion:**

These findings suggest that compared to non-diabetic individuals, diabetic individuals have an increased incidence and mortality of bladder cancer.

## Introduction

Bladder cancer is one of the most common malignancies of the urinary tract. Based on incidence and mortality data from several agencies, the American Cancer Society estimates that 73,510 new bladder cancer cases and 14,880 deaths from bladder cancer are projected to occur in the United States in 2012 [1]. To explore the effective tools for prevention of bladder cancer, great investment has been made to gain new insight into how environmental and genetic factors influence the development of bladder cancer. The chemical and environmental exposures include aromatic amines [2], aniline dyes [3], bitumen [4], nitrites and nitrates [5], paint [6] and arsenic [7], but the most important environmental factor is cigarette smoking [8].

Diabetes mellitus (DM) is considered to be one of the major public health challenges in both industrialized and developing countries [9]. A number of studies have found that diabetes may be associated with increased risk of a variety of cancers, including cancers of the pancreas [10], liver [11], kidney [12], colon and rectal [13]. A previous meta-analysis of 16 studies (seven case-control studies, three cohort studies and six cohort studies of diabetic patients) conducted in 2006 showed that diabetes was associated with an increased risk of bladder cancer in case-control studies and cohort studies, but not in cohort studies of diabetic patients [14]. However, the association between diabetes and mortality from bladder cancer remains unclear. Since the meta-analysis was published, a variety of relevant studies on such association have yielded inconsistent results [15], [16], [17], [18], [19], [20], [21], [22], [23], [24], [25], [26], [27], [28], [29], [30]. Currently, we aim to analyze the relation between DM and incidence and mortality of bladder cancer in an updated meta-analysis of cohort studies. This updated analysis of 29 cohort studies will allow us to provide more precise risk estimates than the previous analysis. We also evaluated whether the association varied by sex, and assessed potential confounders including smoking and obesity.

## Methods

### Data Sources and Searches

A computerized literature search was conducted using MEDLINE (from 1 January 1966) and EMBASE (from 1 January 1974) through 31 March 2012 by two independent investigators. The search strategy used medical subject heading (MeSH) terms and keywords: diabetes or diabetes mellitus; bladder; neoplasm(s) or cancer; mortality; and epidemiologic studies. Diabetes mellitus was determined through medical records, fasting plasma glucose, hospital discharge register or self-reported history of diabetes. Bladder cancer was assessed by cancer registries, medical records, death certificates and ambulatory and inpatient claims. We also manually reviewed the reference lists to identify additional relevant studies. No language restrictions were imposed. Our systematic review was conducted according to the meta-analysis of observational studies in epidemiology (MOOSE) guidelines [31].

### Study Selection

We included those studies that met all of the following criteria: (1) they had a prospective or retrospective cohort design; (2) one of the exposure of interest was DM; (3) one of the outcome of interest was bladder cancer; and (4) reported rate ratio, hazard ratio, or standardized incidence/mortality rate (SIR/SMR) with their 95% confidence intervals (CIs), or provided sufficient information to calculate them. We summarized results from cohort studies because they are less prone to selection bias compared to case-control studies.

Studies were excluded if (1) case–control design was used; or (2) they provided only an effect estimate with no means to calculate a CI. To evaluate studies’ eligibility for inclusion, titles, abstracts, and articles were reviewed independently by two authors; discrepancies were resolved by a third reviewer or by consensus. Articles or reports from non-peer-reviewed sources were not included in this meta-analysis. In the event of multiple publications from the same study population, the most recent publication with the largest number of bladder cancer cases was included in the meta-analysis. We did not consider studies in which the exposure of interest was mainly or solely type 1 diabetes, defined as diagnosis before 30 years of age.

### Data Extraction

Two investigators independently performed the data extraction. When discrepancies were found, a third investigator would make the definitive decision for data extraction. The extracted information included: the first author’s last name, publication year, study location, participant characteristics (age and sex), sample size, measure of association, length of follow-up (if applicable), variables adjusted in the analysis, and the risk estimates with corresponding 95% CIs. From each study, we extracted the RR estimate that was adjusted for the greatest number of potential confounders.

### Statistical Analysis

We divided epidemiologic studies of the relationship between diabetes and risk of bladder cancer into two general types according to the measure of relative risks (RRs): cohort studies (rate ratio or hazard ratio), and cohort studies of diabetic patients using external population comparisons (SIR/SMR). In practice, these four measures of effect yield similar estimates of RR because the absolute risk of bladder cancer is low. We conducted separate meta-analyses of bladder cancer incidence and mortality.

For incidence and mortality of bladder cancer, the study quality was assessed according to the following: evaluation of diabetes, outcome ascertainment, duration of follow-up, loss to follow-up, and number of adjustments [32]. In this meta-analysis, the maximum quality score was 10 points, and studies with quality score greater than or equal to 5 points were considered high quality (Supporting Information **Figure S1** **and S2**).

The variance of the log RR from each study was calculated by converting the 95% CI to its natural logarithm by taking the width of the CI and dividing by 3.92 [10], [32]. Summary relative risk estimates with corresponding 95% CIs were derived using the method of DerSimonian and Laird with the assumptions of a random-effects model, which considers both within-study and between-study variations [33]. When sex-specific estimates were available, we first analyzed together (as RR estimates for bladder cancer) and then separately (as RR estimates for bladder cancer in different gender groups).

To investigate the sources of heterogeneity in relative risk, we performed heterogeneity tests and sensitivity analysis. In assessing heterogeneity among studies, we used the Cochran Q test and I^2^ statistics [34]. This was used to test whether the differences obtained between studies were due to chance. For the Q test, a p value of less than 0.05 was used as an indication of the presence of heterogeneity; for I^2^, a value >50% is considered a measure of severe heterogeneity. To explore the potential heterogeneity between studies, we conducted analyses stratified by study design, geographic region, publication year, gender, and we also evaluated the impact of adjustment for smoking and body mass index (BMI) on the association between diabetes and the risk of bladder cancer. Sensitivity analysis was performed by excluding each study individually to assess its influence on the overall result of the meta-analysis.

Publication bias was evaluated using a funnel plot of a trial’s effect size against the SE. Because funnel plots have several limitations and represent only an informal approach to detect publication bias, we further carried out formal testing using the test proposed by the Begg’s adjusted rank correlation test and by the Egger’s regression test [35], [36]. All statistical analyses were performed using STATA version 11.0 (STATA, College Station, TX, USA). A two-tailed P value of less than 0.05 was considered to be statistically significant.

## Results

### Characteristics in Selected Studies

**Figure 1:**
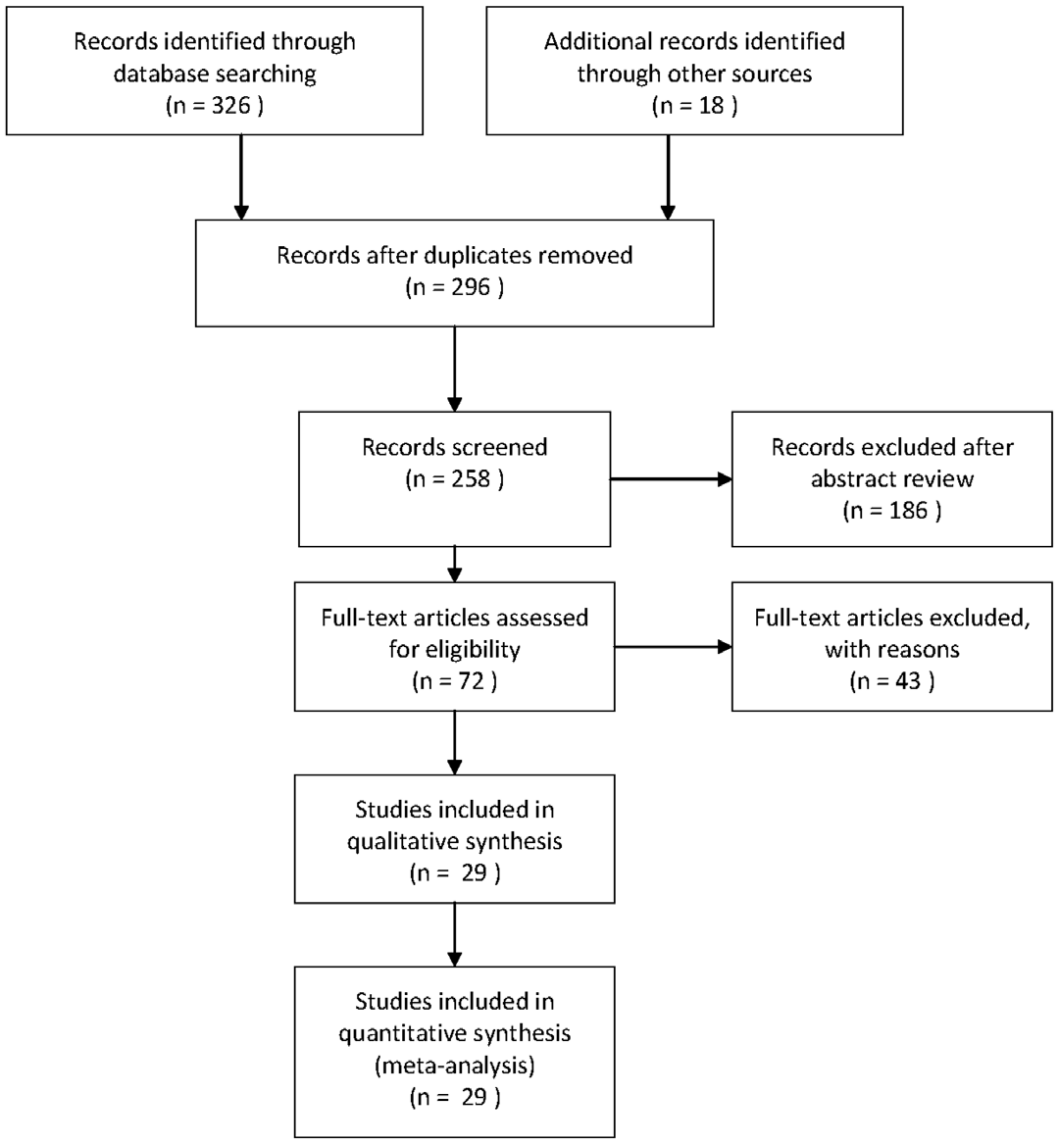
Flow chart on the articles selection process.

The search strategy generated 258 citations, of which 72 were considered of potential value and the full text was retrieved for detailed evaluation (**Figure 1**). Fifty-three of these 72 articles were subsequently excluded from the systematic review. 50 studies did not satisfy inclusion criteria. Two cohort studies were excluded because they presented a relationship between bladder cancer and type 1 DM [37], [38]. Another one study was also excluded because it presented results on an association of DM and mortality of urinary system diseases, but not specific for the association between diabetes and risk of bladder cancer [39]. Additional eight articles were included from reference review. Thus, a total of 27 articles (29 studies), which met the inclusion and exclusion criteria, were used in this meta-analysis.

Of these 29 cohort studies which reported an association between diabetes and risk of bladder cancer, 20 studies employed incidence and/or mortality rates as the measurement of RR (**Table S1**) [40], [41], [42], [16], [18], [19], [20], [21], [23], [43], [15], [17], [22], [44], [25], [26], [27], [28], [29], and nine studies used SIR/SMR as the measurement of RR (**Table S2**) [45], [46], [47], [24], [48], [49], [50], [30]. Eight studies were conducted in North America, 11 in Europe, eight in Asia, and two in multiple countries. The study population in 24 studies consisted of men and women, four studies consisted entirely of men and one study included women only.

DM was determined mainly on the basis of blood glucose levels, medical records, hospital discharge diagnosis in most studies and in four studies, the criteria for DM diagnosis was not indicated clearly [20], [21], [49], [30]. Bladder cancer diagnosis was made by cancer registry or death certificate, except for three studies in which the method of outcome ascertainment was not available [21], [27], [49]. Potential confounders were controlled in most of the studies, except in 10 studies [17], [45], [46], [47], [24], [48], [49], [50], [48,30], the confounders adjusted for were not indicated clearly.

### DM and Bladder Cancer Incidence

**Figure 2:**
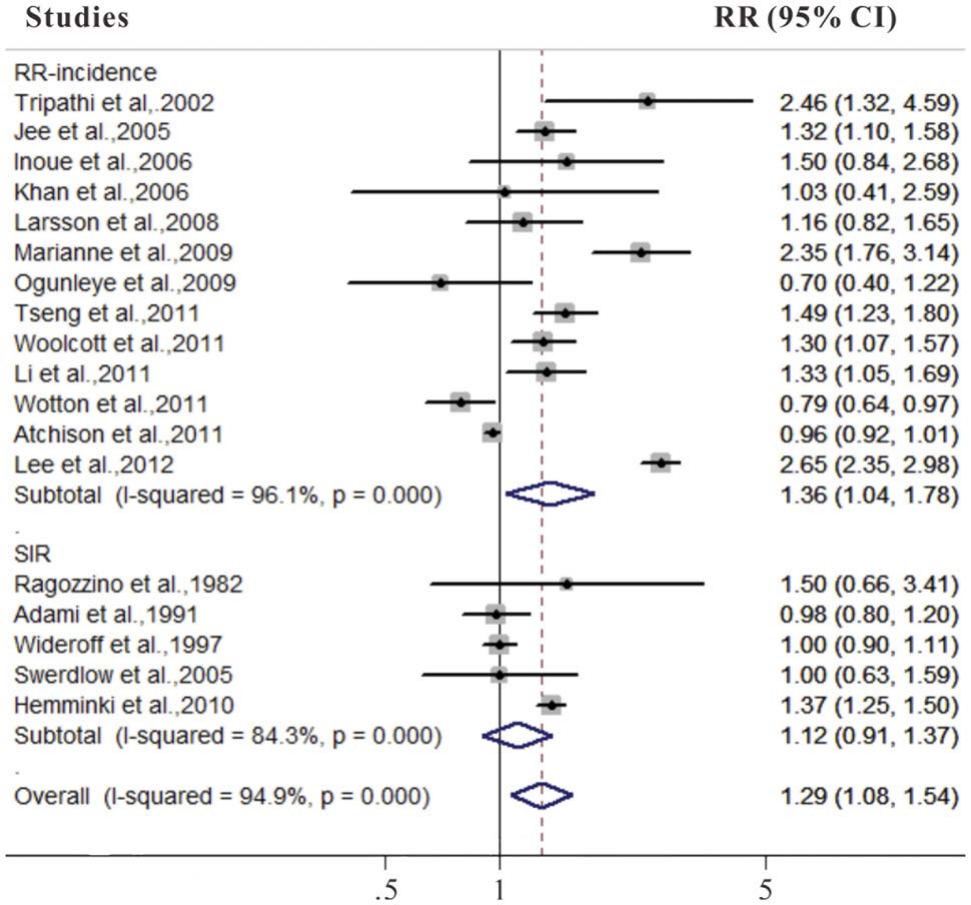
Forest plots of bladder cancer incidence/standard incidence rate associated with diabetes.

We identified 18 cohort studies that reported results on diabetes and bladder cancer incidence (**Tables S1, S2**). As shown in **Figure 2**, the summary RR was 1.29 (95% CI 1.08–1.54) in a random-effects model for diabetic patients, compared with individuals without DM. There was significant heterogeneity among these studies (Q = 331.00, *P*<0.001, *I^2^* = 94.9%).

In a sensitivity analysis in which we removed one study at a time and analyzed the rest, the RRs ranged from 1.32 (95% CI 1.09–1.60) after excluding the study by Atchison et al. [15] (the study which carried the most weight) to 1.30 (95% CI 1.08–1.56) after excluding the study by Khan et al. [42] (the study which carried the least weight).

In analysis stratified by study design, the summary RR was 1.36 (95% CI 1.04–1.78) in cohort studies. However, diabetes was not associated with bladder cancer incidence in cohort studies of diabetic patients (RR 1.12, 95% CI 0.91–1.37). The summary estimates were significantly higher for studies conducted in Asia and North America than in Europe, and for studies published in 2006 or later than for studies published before 2006. The positive association between diabetes and bladder cancer incidence was observed in the men (RR 1.36, 95% CI 1.05–1.77), but not in the women (RR 1.28, 95% CI 0.75–2.19) (**Table 1**).

**Table 1:** Summary relative risks for the association between diabetes and bladder cancer incidence/SIR.

Subgroup	No. of studies	RR (95% CI)	Tests	for	heterogeneity
			Q	*P*	*I^2^* (%)
Study design					
DM-free as controls	13	1.36 (1.04–1.78)	304.98	0.000	96.1
Population as controls	5	1.12 (0.91–1.37)	25.53	0.000	84.3
Geographical region					
Europe	7	1.01 (0.84–1.22)	40.61	0.000	85.2
North America	6	1.49 (1.08–2.05)	57.41	0.000	91.3
Asia	5	1.61 (1.09–2.38)	54.40	0.000	92.6
Publication year					
1970–2005	6	1.16 (0.96–1.40)	15.41	0.009	67.6
2006–2012	12	1.32 (1.03–1.69)	311.80	0.000	96.5
Gender					
Male	10	1.36 (1.05–1.77)	190.22	0.000	95.3
Female	6	1.28 (0.75–2.19)	78.04	0.000	93.6
Adjustment for smoking					
Yes	7	1.33 (1.19–1.47)	4.83	0.565	0.0
No	11	1.24 (0.98–1.58)	318.26	0.000	96.9
Adjustment for BMI, yes					
Yes	5	1.30 (0.95–1.77)	17.52	0.002	77.2
No	13	1.28 (1.03–1.59)	216.39	0.000	94.5

*RR* relative risk, *CI* confidence interval, *DM* diabetes mellitus, *BMI* body mass index.

We also investigated the impact of confounding factors on the estimates of relative risk. When we restricted the meta-analysis to those studies controlled for smoking, the positive association between diabetes and bladder cancer incidence remained (RR 1.33, 95% CI 1.19–1.47). The summary estimates were similar for studies that adjusted for BMI and for studies that did not. There was statistically significant heterogeneity within most subgroups.

### DM and Bladder Cancer Mortality

**Figure 3:**
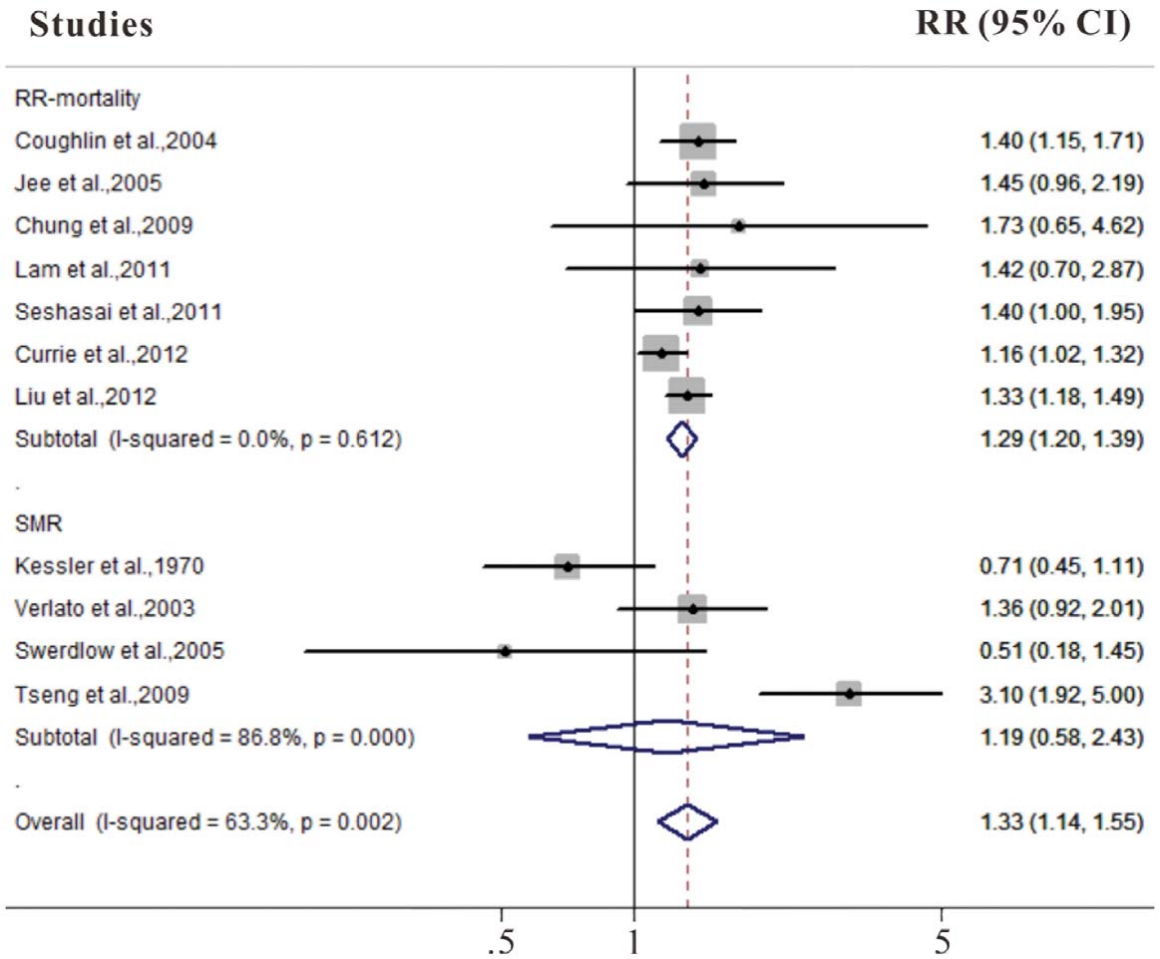
Forest plots of bladder cancer mortality/standard mortality rate associated with diabetes.

We identified 11 cohort studies that presented results on diabetes and mortality from bladder cancer (**Tables S1, S2**). As shown in **Figure 3**, the summary RR was 1.33 (95% CI 1.14–1.55) in a random-effects model for diabetic patients, compared with individuals without DM. There was significant heterogeneity among these studies (Q = 27.26, *P* = 0.002, *I^2^* = 63.3%).

In the sensitivity analysis, our analysis confirmed the stability of the positive association between DM and mortality from bladder cancer. For example, when we excluded the study of Liu et al. [28] from the analysis (this was the study that clearly carried the most weight), the estimated pooled RR was similar (RR 1.33, 95% CI 1.09–1.63), with significant heterogeneity (*P* = 0.001, *I^2^* = 66.5%).

In analysis stratified by study design, the summary RR was 1.29 (95% CI 1.20–1.39) in cohort studies. However, diabetes was not associated with mortality from bladder cancer in cohort studies of diabetic patients (RR 1.19, 95% CI 0.58–2.43). The summary estimates were higher for studies conducted in Asia than in other region and for studies published in 2006 or later than for studies published before 2006. Three studies provided results on cancer mortality specific for gender; additional two studies consisted entirely of men. Diabetes was associated with an increased mortality from bladder cancer in both males and females (RR = 1.54, 95% CI 1.30–1.82 in males, and RR = 1.50, 95% CI 1.05–2.14 in females, respectively) (**Table 2**).

**Table 2:** Summary relative risks for the association between diabetes and bladder cancer mortality/SMR.

Subgroup	No. of studies	RR (95% CI)	Tests	for	heterogeneity
			Q	*P*	*I^2^* (%)
Study design					
DM-free as controls	7	1.29 (1.20–1.39)	4.48	0.612	0.0
Population as controls	4	1.19 (0.58–2.43)	22.75	0.000	86.8
Geographical region					
North America	4	1.03 (0.53–2.00)	7.35	0.007	86.4
Europe	2	1.25 (1.15–1.36)	5.38	0.146	44.2
Asia	3	1.98 (1.47–2.66)	5.64	0.060	64.5
Other^a^	2	1.40 (1.04–1.89)	0.00	0.971	0.0
Publication year					
1970–2005	5	1.15 (0.85–1.55)	10.81	0.029	63.0
2006–2012	6	1.45 (1.18–1.78)	16.39	0.006	69.5
Gender					
Men	5	1.54 (1.30–1.82)	7.34	0.119	45.5
Women	3	1.50 (1.05–2.14)	2.44	0.296	17.9
Adjustment for smoking					
Yes	5	1.29 (1.19–1.39)	4.06	0.398	1.5
No	6	1.30 (0.77–2.18)	23.05	0.000	78.3
Adjustment for BMI					
Yes	3	1.35 (1.23–1.49)	0.24	0.888	0.0
No	8	1.31 (0.97–1.76)	25.1	0.001	72.1

*RR* relative risk, *CI* confidence interval, *DM* diabetes mellitus, *BMI* body mass index.

a One study conducted in Asia-Pacific region, the other study conducted in North America, Europe, Japan and other region.

When we restricted the meta-analysis to those studies controlled for smoking, a significant positive association was found between diabetes and mortality from bladder cancer (RR 1.29, 95% CI 1.19–1.39). The summary estimates were also significantly higher for studies that reported BMI-adjusted RRs than for those which did not [RR (95% CI): 1.35 (1.23–1.49) versus 1.31 (0.97–1.76)].

### Publication Bias

**Figure 4:**
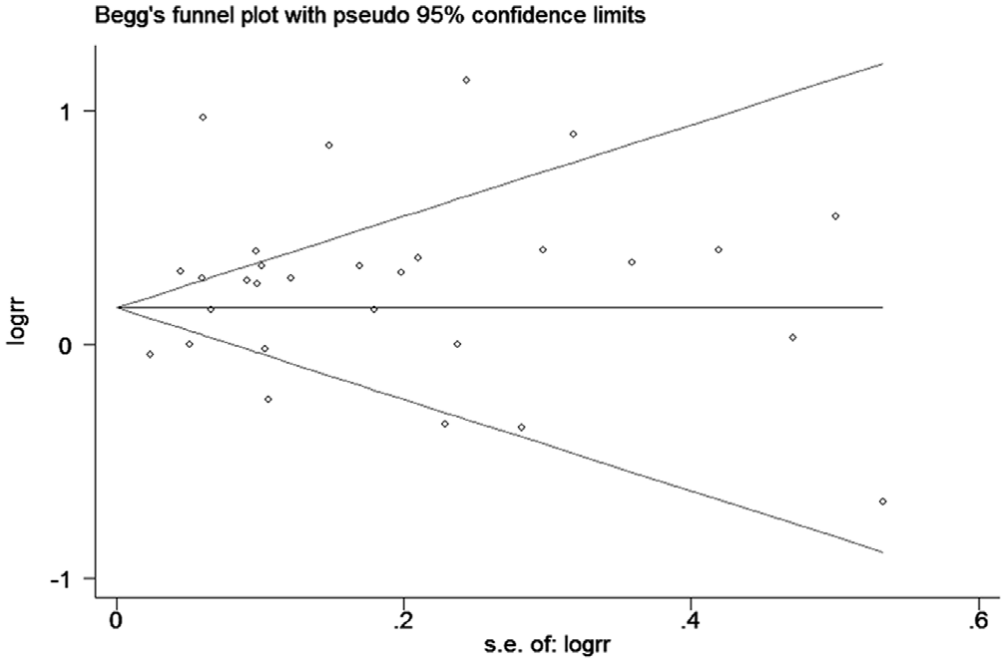
Funnel plot of cohort studies evaluating the association between diabetes and bladder cancer risk.

There was no funnel plot asymmetry for the association between DM and risk of bladder cancer. P values for Begg’s adjusted rank correlation test was 0.268 and the Egger’s regression asymmetry test was 0.139, suggesting a low probability of publication bias (**Figure 4**).

## Discussion

Findings of this meta-analysis of cohort studies indicated that compared with non-diabetics or general population, individuals with diabetes may have more than 29% increased incidence of bladder cancer. However, the positive association was only observed in men. Of note, diabetes is associated with an increased mortality from bladder cancer in both males and females.

The previous meta-analysis has evaluated the association of diabetes and risk of bladder cancer. Most studies included in the meta-analysis were performed in Western countries, and only one study was conducted in the Asian population in Korea [14]. Moreover, the association in different gender groups is worthy of investigation, but has not been looked at. On account of the dismal prognosis, this meta-analysis extracted mortality rate as a substitution for incidence rate in several studies. However, the association between diabetes and mortality from bladder cancer is unclear.

Our study extends previous meta-analysis by providing a more precise estimate of the association between diabetes and risk of bladder cancer risk (based on 29 cohort studies). Case-control studies are susceptible to recall and selection biases which might inflate the RRs. Most of the included original studies were prospective, which probably do not reduce so much the possibility of reverse causation if a rather long time interval (at least 1 year) between inclusion and cancer diagnosis is not required. Moreover, we also investigated the impact of confounding factors, including smoking and BMI, on the estimates of relative risk.

Studies investigating the association between diabetes mellitus and cancer have reported inconsistent findings. Ogunleye and colleagues found that significantly increased risks were only observed for pancreatic, liver and colon cancer [20]. Wotton et al. demonstrated that diabetes mellitus was associated with an elevated risk of cancers of the liver, pancreas and uterus [23]. It has also been suggested that DM is a cancer preventive agent in some studies. There were significantly low rate ratios for cancer of the prostate and non-melanoma skin cancer in people admitted to hospital for diabetes mellitus when aged 30 or older [23]. Zhang et al. also observed that diabetes mellitus was associated with decreased incidence of prostate cancer, specifically in the population of the United States [51]. In overweight and obese patients with type 2 diabetes, lower androgen levels could explain a potentially protective effect against prostate cancer [52].

These inconsistent results may be due to different study population and follow-up periods. Furthermore, therapeutic agents used in the treatment have been also implicated in altering cancer risk [53], [54]. Metformin therapy in diabetic patients appears to be associated with a significantly lower risk of cancer incidence and mortality [55], [56], [57]. Colmers et al. found that use of thiazolidinediones (TZDs) was associated with a modest but significantly decreased risk of lung, colorectal and breast cancers [58]. However, TZDs were associated with an increased risk of bladder cancer among adults with type 2 diabetes [59]. The important outstanding prognostic question is whether diabetes mellitus is associated with an increased or a reduced risk of certain cancer. Noteworthy, it is possible to draw more accurate conclusions in a larger cohort of diabetic patients by conducting meta-analysis of the studies published on the subject.

This study has several limitations which should be recognized. First, cohort studies, which may not be prone to recall bias but are prone to selection bias because patients with diabetes are under increased medical surveillance. This bias may distort the true effects.

Second, diabetes in some studies was based on self-report, which may result in misclassification of diabetic persons as non-diabetic persons. This underestimation would tend to attenuate any true association between diabetes and risk of bladder cancer.

Third, great heterogeneity exists in terms of study design, geographical region, publication year, gender and adjustment for confounding factors. Despite the use of appropriate meta-analytic techniques with random-effect models, we could not account for these differences.

Fourth, most studies included in this meta-analysis did not consider the role of anti-diabetic drugs in bladder cancer. For example, increasing studies have suggested that use of pioglitazone (a common anti-diabetic drug) was associated with an increased incidence of bladder cancer [60], [61]. In our meta-analysis, the association between diabetes and incidence of bladder cancer was weaker in women than in men. Neumann observed a significant association between pioglitazone and bladder cancer for men but not women [62]. Noteworthy, pioglitazone use might be also involved in such a sex discrepancy. However, only a few studies adjusted for anti-diabetic drugs. Fifth, confounding cannot be fully excluded as a potential explanation for the observed association, because these two diseases share several risk factors, such as smoking and obesity. In our meta-analysis, adjustment for smoking and BMI significantly alter the relationship between diabetes and risk of bladder cancer.

Finally, inherent in any meta-analysis of published data is the possibility of publication bias, that is small studies with null results tend not to be published. Publication bias may have resulted in an overestimate of the relationship between DM and risk of bladder cancer. However, the results obtained from funnel plot analysis and formal statistical tests did not provide evidence for such bias.

The possible mechanisms underlying the association of diabetes with bladder cancer risk are still uncertain. Insulin has been hypothesized to be a cancer growth promoter which could explain an increased cancer risk in adults with type 2 diabetes [63], [64]. Elevated insulin concentrations would lower concentrations of IGF-binding proteins (IGFBPs), which in turn contribute to an up-regulated level of IGFs. In the circulation, IGF-I binds mainly to IGFBP-3 and stimulates cell proliferation and inhibits apoptosis [65]. Emerging evidence indicates that IGF-I and IGFBP-3 may also play a role in the development of bladder cancer. In a US case-control study, patients with bladder cancer have higher plasma levels of IGF-1 and lower levels of IGFBP-3 than controls [66]. Dunn and colleagues demonstrated that IGF-I could contribute to bladder carcinogenesis in animal studies [67]. Moreover, a lower preoperative plasma IGFBP-3 level was associated with metastases to regional lymph nodes, bladder cancer progression, and survival [68].

Although the absolute risk of bladder cancer is low among diabetic individuals, our results have important clinical and public health significance. As a serious and growing health problem in USA, DM affects nearly 25.8 million children and adults in the United States, 8.3% of the U.S.population in 2010 (http://www.diabetes.org/diabetes-basics/diabetes-statistics/). In China, the age-standardized prevalences of total diabetes and prediabetes among Chinese adults (20 years of age or older) are 9.7 and 15.5%, respectively [69]. Due to growing obesity epidemic, the prevalence of diabetes will probably increase and contribute to the risk of bladder cancer among diabetic patients. In patients with non-muscle invasive bladder cancer, DM was found to be an independent factor for recurrence- and progression-free survival [70]. For diabetic patients undergoing radical cystoprostatectomy and ileal orthotopic bladder substitution, it takes longer to regain daytime and nighttime continence than nondiabetic patients [71]. In clinical practice, the diabetic patients should be informed of the potential negative impact of DM on disease recurrence, progression, survival and the recovery of urinary continence after an ileal orthotopic bladder substitution.

In conclusion, this meta-analysis supports the hypothesis that diabetic individuals have an increased incidence of bladder cancer. Further analysis indicates that the positive relation is observed only in the men, but not in the women. However, diabetes is associated with an increased mortality from bladder cancer in both males and females. More research, both epidemiological and mechanistic, is warranted to clarify the association between diabetes and risk of bladder cancer.

## Supporting Information

Figure S1 **Quality scores of cohort studies of diabetes and bladder cancer risk based on rate/hazard ratio.**
(TIF)

Figure S2 **Quality scores of cohort studies of diabetes and bladder cancer risk based on standardized incidence/mortality ratio.**
(TIF)

Table S1 **Characteristics of 20 cohort studies of diabetes and bladder cancer risk based on rate/hazard ratio.**
(DOC)

Table S2 **Characteristics of 9 cohort studies of diabetes and bladder cancer risk based on standardized incidence/mortality ratio.**
(DOC)

Diagram S1 **PRISMA (Preferred Reporting Items for Systematic Reviews and Meta-Analyses) flow diagram.**
(DOC)

Checklist S1 **PRISMA (Preferred Reporting Items for Systematic Reviews and Meta-Analyses) checklist.**
(DOC)
