# Supplementary figures and images for: Diabetes Mellitus and Risk of Bladder Cancer: A Meta-Analysis of Cohort Studies
Source: PLoS One. 2013 Feb 20;8(2):e56662. doi: 10.1371/journal.pone.0056662 (PMC3577653; doi:10.1371/journal.pone.0056662)

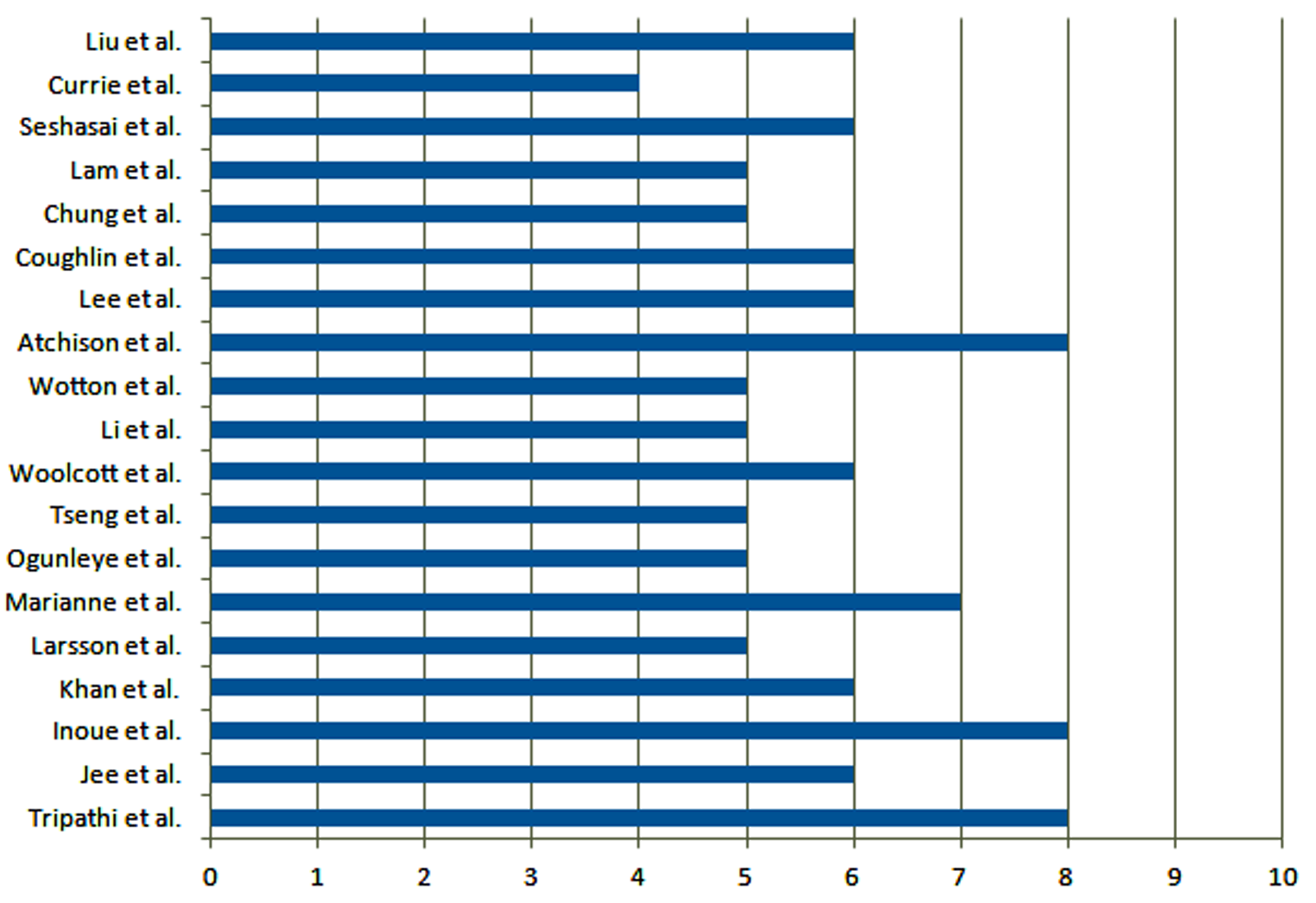

Supplement: Figure S1 — Quality scores of cohort studies of diabetes and bladder cancer risk based on rate/hazard ratio. (TIF) [file pone.0056662.s001.tif]

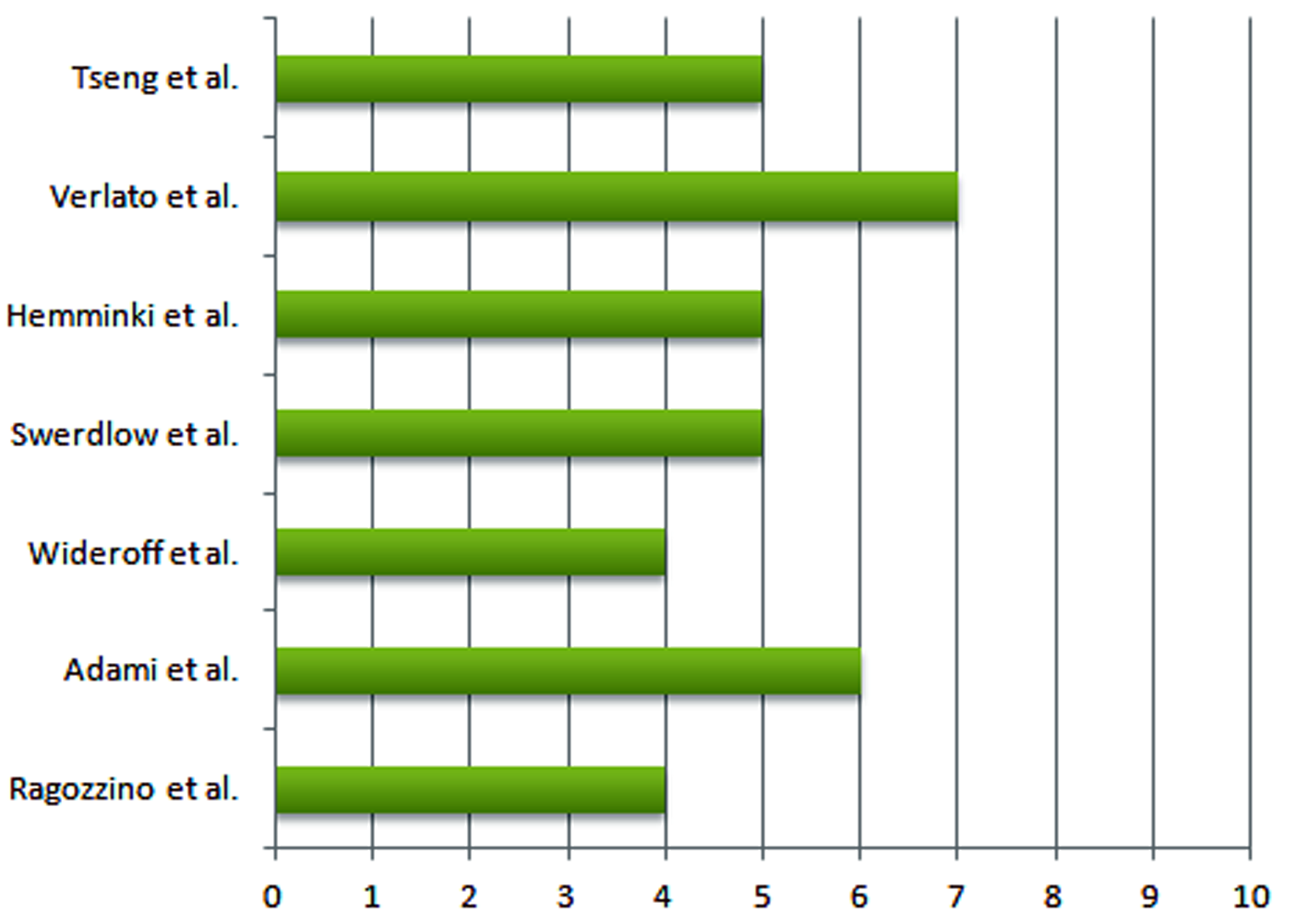

Supplement: Figure S2 — Quality scores of cohort studies of diabetes and bladder cancer risk based on standardized incidence/mortality ratio. (TIF) [file pone.0056662.s002.tif]
