# Supplementary material for: Diabetes Mellitus and Risk of Bladder Cancer: A Meta-Analysis of Cohort Studies
Source: PLoS One. 2013 Feb 20;8(2):e56662. doi: 10.1371/journal.pone.0056662 (PMC3577653; doi:10.1371/journal.pone.0056662)
Supplement: Table S1 — Characteristics of 20 cohort studies of diabetes and bladder cancer risk based on rate/hazard ratio. (DOC) [file pone.0056662.s003.doc]

**Table S1 Characteristics of 20 cohort studies of diabetes and bladder cancer risk based on rate/hazard ratio**

| Author/(country) | No. of  subjects | Demographics  of all subjects  (age, years) | Diabetes  assessment | Bladder cancer assessment | Follow  up,  years | Adjusted RR  (95% CI) | Adjustments |
| --- | --- | --- | --- | --- | --- | --- | --- |
| **Incidence** |  |  |  |  |  |  |  |
| Tripathi et  al.(USA) | 37,459 | Age: NA  Female: 100% | Self-reported | Cancer  registries | 13 | 2.46 (1.32-4.59)(f) | Age, smoking, physical activity,  BMI, alcohol, occupation, marital  status |
| Jee et al.  (Korea) | 1,298,385 | Age: 47  Male: 64% | Self-reported or  FGP | Cancer registry  and  hospitalization  records | 10 | 1.32 (1.10, 1.57)(m) | Age, age squared, smoking, alcohol |
| Inoue et al.  (Japan) | 97,771 | Age: 40-69  Male: 48% | Self-reported | Cancer  registries and  hospitalization  records | 10.7 | 1.63 (0.89-3.0) (m)  0.64 (0.09-4.75) (f)  1.5 (0.84-2.69)(both) | Age, study area, smoking, alcohol,  BMI, physical activity, green  vegetable and coffee intake,  history of cerebrovascular or  ischemic heart disease |
| Khan et al.  (Japan) | 56,881 | Age: 40-79  Male: 41% | Self-reported | Cancer  registries and  death  certificates | 8 | 1.03 (0.41-2.60)(m) | Age, BMI, smoking, alcohol |
| Larsson et  al.(Sweden) | 45,906 | Age: 45-79  Male: 100% | Self-reported | Cancer  registries | 9.3 | 1.16 (0.81–1.64)(m) | Age, education, smoking |
| Marianne et  al. (USA) | 442,712 | Age: 54  Male: 52% | Medical records  or oral  antidiabetic  agent | Medical  records | 3.5 | 2.35 (1.76-3.15) | Age, sex, schistosomiasis, pelvic  radiation |
| Ogunleye et  al. (UK) | 28,731 | Age: 62  Male: 53.3% | NA | Cancer  registries | 4 | 0.7 (0.4-1.21) | Deprivation decile |
| Tseng et al.  (Taiwan) | 998,947 | Age: NA  Male: 49.6% | NA | NA | 3 | 1.49 (1.23-1.80) | Age, sex, living regions,  occupation, systematic disease  and oral drugs |
| Woolcott et  al. (USA) | 185,816 | Age: 60.4  Male: 48.1% | Self-reported | Cancer  registry and  surveillance  and national  death index | 10.7 | 1.30 (1.07-1.57) | Smoking status, intensity and  duration, and employment in a  high risk industry |
| Li et al.  (USA) | 397,783 | Age: 46.8  Male: 38.2% | Self-reported | Self-reported | NA | 1.7 (1.2-2.2)(m)  0.9 (0.6–1.3)(f)  1.33 (1.05-1.69)(both) | Age, race/ethnicity, health  insurance coverage, smoking  status, heavy drinking, BMI,  physical inactivity |
| Wotton et  al.(England) | 484,356 | Age: ≥30  Male: 54% | Medical records | Death  certificates | NA | 0.79 (0.64-0.97) | Age, sex, time period in single  calendar years, district of  residence |
| Atchison et  al. (USA) | 4,501,578 | Age: 59.1  Male: 100% | Hospital  discharge  diagnosis | Hospital  admission  diagnosis | 11.7 | 0.96 ( 0.92-1.01) | Age, time, latency, race and  number of visits, diagnoses of  alcohol-related conditions, obesity  and COPD |
| Lee et al.  (Taiwan) | 985,818 | Age: NA  Male: 49.6% | Ambulatory and  inpatient claims | Ambulatory  and inpatient  claims | 12 | 2.77 (2.37-3.22)(m)  2.53 (2.09-3.07)(f)  2.65 (2.35-2.98)(both) | NA |
| **Mortality** |  |  |  |  |  |  |  |
| Coughlin et  al. (USA) | 1,056,243 | Age: 57  Male: 44% | Self-reported | Death  certificates | 16 | 1.43 (1.14-1.8)(m)  1.3 (0.85-2.0)(f)  1.4 (1.15-1.71)(both) | Age, race, years of education, BMI,  smoking, alcohol, total red meat  consumption, consumption of  citrus fruits, juices and  vegetables, physical activity, use  of replacement estrogens |
| Jee et al.  (Korea) | 1,298,385 | Age: 47  Male: 64% | Self-reported or  FGP | Cancer registry  and  hospitalization  records | 10 | 1.45 (0.96, 2.19)(m) | Age, age squared, smoking, alcohol |
| Chung et al.  (Taiwan) | 54,751 | Age: 40-80  Male: 100% | FGP and  self-reported | Death registry | 6.8 | 1.22 (0.27-5.61) | Age |
| Lam et al.  (Asia,  Australia) | 367,361 | Age: 48  Male: 59% | Self-reported or  blood glucose  level | NA | 4.0 | 1.42 (0.70, 2.86) | Age |
| Seshasai et  al. (Europe,  North  America,  Japan, other) | 820,900 | Age: 55  Male: 52% | Self-reported,  FPG,  medication use | Death  certificates | 13.6 | 1.4 (1.01-1.96) | Age, smoking, BMI |
|  |  |  |  |  |  |  |  |
| Currie et al.  (UK) | 112,408 | Age: 71.4  Male: 48.1% | Read code  indicative of  diabetes | General  practice  routine data | 2.0 | 1.16 (1.02-1.32) | Age at baseline, sex, smoking,  Charlson comorbidity index, year  of diagnosis |
| Liu et al.  (Sweden) | 1,016,105 | Age: 67.0  Male: NA | Hospital  discharge  register | Cancer  registry | 8 | 1.33 (1.18-1.49) | Age at diagnosis, sex, period,  obesity, alcohol, smoking,  socioeconomic status, and  diagnosis region |

*RR* relative risk, *CI* confidence interval, *COPD* chronic obstructive pulmonary disease, *m* male, *f* female, *BMI* body mass index, *NA* data not applicable, FPG fasting plasma glucose
