# Supplementary material for: Diabetes Mellitus and Risk of Bladder Cancer: A Meta-Analysis of Cohort Studies
Source: PLoS One. 2013 Feb 20;8(2):e56662. doi: 10.1371/journal.pone.0056662 (PMC3577653; doi:10.1371/journal.pone.0056662)
Supplement: Table S2 — Characteristics of 9 cohort studies of diabetes and bladder cancer risk based on standardized incidence/mortality ratio. (DOC) [file pone.0056662.s004.doc]

**Table S2 Characteristics of 9 cohort studies of diabetes and bladder cancer risk based on standardized incidence/mortality ratio**

| Author/ (country) | No. of  subjects | Demographics  (age, years) | Diabetes  assessment | Bladder cancer assessment | Follow  up,  years | Adjusted RR (95% CI) |
| --- | --- | --- | --- | --- | --- | --- |
| **Incidence** |  |  |  |  |  |  |
| Ragozzino et al.  (USA) | 1,135 | NA | Medical records | Death certificates, autopsy reports,  hospital records, outpatient visits | 8.6 | 1.49 (0.6-3.07) |
| Adami et al. (Sweden) | 51,008 | Age: NA  Male: 45.4% | Discharge diagnosis | Cancer Registry | 5.2 | 1.0 (0.8-1.3)(m)  0.9 (0.6-1.3)(f)  0.98 (0.8-1.2)(both) |
| Wideroff et al.  (Denmark) | 109,581 | Age: 66.5  Male: 49.8% | Discharge records | Cancer registry | 5.7 | 1.0 (0.9-1.1)(m)  0.9 (0.8-1.1)(f)  1.0 (0.9-1.1)(both) |
| Swerdlow et al.  (UK) | 28,900 | Age: ＜50  Male: 54.3% | National and geographical registers | Cancer Register | 18 | 1.0 ( 0.61 –1.55) |
| Hemminki et al.  (Sweden) | 125,126 | Age: ＞39  Male: NA | Hospital Discharge Register | Cancer Registry | 15 | 1.37 (1.25-1.49) |
| **Mortality** |  |  |  |  |  |  |
| Kessler et al. (USA) | NA | NA | NA | NA | NA | 0.71 (0.44-1.08) |
| Swerdlow et al.  (UK) | 28,900 | Age: ＜50  Male: 54.3% | National and geographical registers | Cancer Register | 18 | 0.51 ( 0.18 –1.46) |
| Verlato et al. (Italy) | 7,148 | Age: 66.6  Male: 47.1% | Diabetes clinic, family physicians and drug  prescription database | Death certificates | 10 | 1.33 (0.83–2.01)(m)  1.50 (0.55–3.26)(f)  1.36(0.91–1.98)(both) |
| Tseng et al. (Taiwan) | 244,920 | Age: ≥25  Male: 46.3% | NA | National Register of deaths | 12 | 2.93 (2.58-3.33)(m)  2.95 (2.49-3.5)(f)  2.94 (2.65-3.25)(both) |

*RR* relative risk, *CI* confidence interval, *m* male, *f* female, *NA*, data not applicable
