# Supplementary material for: Diabetes Mellitus and Risk of Bladder Cancer: A Meta-Analysis of Cohort Studies
Source: PLoS One. 2013 Feb 20;8(2):e56662. doi: 10.1371/journal.pone.0056662 (PMC3577653; doi:10.1371/journal.pone.0056662)
Supplement: Diagram S1 — PRISMA (Preferred Reporting Items for Systematic Reviews and Meta-Analyses) flow diagram. (DOC) [file pone.0056662.s005.doc]

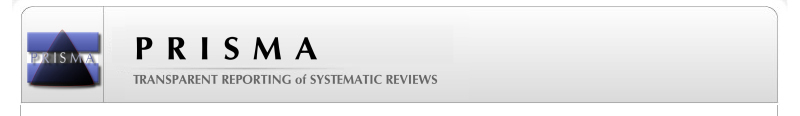
**PRISMA 2009 Flow Diagram**

**Screening**

**Included**

**Eligibility**

**Identification**

Records identified through database searching
(n = 326 )

Additional records identified through other sources
(n = 18 )

Records after duplicates removed
(n = 296 )

Records screened
(n = 258 )

Records excluded after abstract review
(n = 186 )

Full-text articles assessed for eligibility
(n = 72 )

Full-text articles excluded, with reasons
(n = 43 )

Studies included in qualitative synthesis
(n = 29 )

Studies included in quantitative synthesis (meta-analysis)
(n = 29 )
